# Relationship of Serum Uric Acid Level with Demographic Features, Risk Factors, Severity, Prognosis, Serum Levels of Vitamin D, Calcium, and Magnesium in Stroke

**DOI:** 10.1155/2018/6580178

**Published:** 2018-07-02

**Authors:** Payam Saadat, Alijan Ahmadi Ahangar, Mansor Babaei, Mandana Kalantar, Mohammad Ali Bayani, Hiva Barzegar, Hemmat Gholinia, Farbod Zahedi Tajrishi, Sekineh Faraji, Fatemeh Frajzadeh

**Affiliations:** ^1^Mobility Impairment Research Center, Health Research Institute, Babol University of Medical Sciences, Babol, Iran; ^2^Department of Internal Medicine, School of Medicine, Babol University of Medical Sciences, Babol, Iran; ^3^Clinical Research Development Center, Ayatollah Rohani Hospital, Babol University of Medical Sciences, Babol, Iran

## Abstract

**Introduction:**

Stroke is one of the most common neurological disorders with high mortality rates. A large financial burden is imposed on the families and health systems of countries in addition to the problems related to the disabilities caused by the disease for the patients. Extensive research is being conducted on the disease, including studies seeking possible relationships between some biomarkers such as uric acid and stroke.

**Methods:**

This descriptive-analytic cross-sectional study was conducted on 170 stroke patients at Babol Ayatollah Rohani Hospital during 2015-2016. Serum uric acid (SUA) levels were measured and recorded at admission time. Patients' demographic data as well as the stroke type and some of their risk factors were entered in a checklist. The data were analyzed by SPSS.v.23 using chi-square and logistic regression tests. *P* < 0.05 was considered as significant in all analyses.

**Results:**

Of the total 170 included patients, 57% had normal, 25% had low, and the remaining patients (18%) had high SUA levels. There was no significant difference in SUA levels in different types of stroke in both genders. Diabetic ischemic embolic patients had higher levels of SUA than diabetic ischemic thrombotic cases. Patients with low magnesium levels had higher rate of low levels of SUA in ischemic stroke.

**Conclusion:**

Serum uric acid levels are not associated with stroke types and gender. Diabetic embolic ischemic stroke cases had high SUA levels than thrombotic types and in ischemic stroke patients with low serum levels of magnesium, SUA levels were also lower.

## 1. Introduction

Stroke is one of the most common neurological disorders, recognized as the third most common cause of death after cancer and cardiovascular disease in developed countries [1].

Stroke patients who survive often have severe disabilities and need special care and long-term rehabilitation; that is why stroke is among the most debilitating neurological diseases in adults [2].

Consequently, in addition to high mortality rate, stroke is one of the most important healthcare issues in the world, due to many financial burdens imposed on families and the countries.

As the classic risk factors of stroke have been identified and preventive measures have been taken to eliminate them, the incidence of stroke has decreased in recent years [3]. Investigating other possible risk factors of stroke is highly important and there is a lot of research in this field. A group of these studies are about possible relationships between some biomarkers such as uric acid and stroke [4].

Uric acid is produced from Purine decomposition in the body. Purine amino acids can be found in several high-protein foods. Uric acid enters the blood stream after production and when passing through the kidneys, it is excreted into the urine. The serum levels of uric acid can be high or low under different conditions. High uric acid may not be symptomatic in conditions such as obesity, high-fat protein diets, alcohol intake, diuretic consumption, some genetic disorders, hypothyroidism, psoriasis, Hodgkin's lymphoma, or renal failure.

Conditions that result in low levels of uric acid in the blood are less common. These include factors such as liver and kidney disorders, frequent contact with chemicals, and genetic abnormalities [5]. Uric acid has strong antioxidant properties, such that 50% of the antioxidant properties of the blood are derived from uric acid [6]. Some studies have recently reported a significant correlation between the reduction in the incidence of stroke and high serum uric acid (SUA) level. Some others have reported controversial findings on the relationship between high or low SUA levels and stroke [7, 8].

Given these issues, there is controversy about any relationship between uric acid and stroke, even if there is this relationship, there is vagueness about the association between uric acid with some variables of stroke such as stroke subtypes, demographic characteristics of patients, severity of stroke at admission and discharge time, stroke risk factors, and some associated variables such as serum magnesium, calcium, and Vit D level in stroke patients. That is why we designed and implemented this study to measure serum uric acid (SUA) levels in stroke patients.

In the case of the existence of any relationships between uric acid and stroke, and specifically with some of its related variables in this study and its confirmation in other studies, it may be possible to use these results in the preventive and therapeutic modalities of these patients.

## 2. Materials and Methods

This cross-sectional descriptive-analytic study was conducted on consecutive stroke patients referred to Ayatollah Rohani Hospital in Babol, Iran, during a one-year period: 2015-2016.

Ayatollah Rouhani Hospital is the main medical center for stroke management in Babol and its surrounding areas.

Ethical approval was obtained prior to the initiation of the study from the ethics committee of Babol University of Medical Sciences (30/1/2076-3385).

Informed consent was also obtained from each participant or their next of kin before any interview or neurologic examination was conducted.

The sample size was designed based on the incidence of stroke in the region [9] and the sample size formula.

The diagnosis of stroke and its types was made according to the criteria in epidemiological studies of stroke [1, 10]. The stroke in this study was divided into ischemic and hemorrhagic types; the ischemic type was divided into thrombotic and embolic and the hemorrhagic type stroke into Intracerebral Hemorrhage (ICH) and Subarachnoid Hemorrhage (SAH) subtypes [11]; given these criteria in this study ischemic embolic stroke included all embolic of cardiac or arterial origin. Brain imaging was performed for all patients and a definitive diagnosis of all stroke cases was confirmed by the neurologist responsible to do the project.

Stroke severity was determined on the basis of NIH Stroke Scale (NIHSS) criteria [12], score ≤ 8 mild stroke: 9–15, moderate stroke: and ≥ 16 severe. All patients who had been diagnosed with any type of stroke for the first time were included in the study (Transient Ischemic Attacks were not included).

Cases that had a history of stroke in the past and those with metabolic, systemic, traumatic, space occupying lesions with hemiparesis or any focal neurological symptoms or signs or stroke mimickers (seizure, migraine) were excluded. Those who had renal or hepatic failure and gout and the ones treated with corticosteroid were also excluded.

The serum uric acid (SUA) levels were measured along with other laboratory tests in the clinical laboratory of Ayatollah Rohani Hospital in Babol.

Serum uric acid level measurements, along with other emergency routine labs, were measured at the time of admission. The maximum interval between the onset of the disease and the time of admission for these patients was up to 12 hours. Serum uric acid levels of 3.4 to 7 and 2.4 to 6 mg/dL were considered as reference normal values for men and women, respectively. The levels were determined by ultrasound enzyme (uricase) PAP chromatography with COBAS (Roche diagnostic, Switzerland).

The measured levels were then entered into a checklist along with the demographic and some other associated factors such as type of the living area (rural-urban) characteristics of the patients.

Other data, including time from symptom onset to hospital arrival, stroke type and its severity at the time of admission and discharge, the results of the laboratory tests performed during admission such as CBC, ESR, CRP, BS, TG, cholesterol, and some other associated factors such as serum magnesium level, serum Ca levels, and serum VitD levels, were entered into the checklist. Having stroke risk factors such as hypertension, ischemic heart disease, diabetes mellitus, hyperlipidemia, and smoking were also entered into the checklist.

At the time of this study, it was not possible to perform intravenous thrombolytic therapy or any interventional procedures at the mentioned center, although these facilities are now available

### 2.1. Data Analysis

The data were analyzed quantitatively and qualitatively using the SPSS (version 23). Chi-square test was used to determine the relationship between serum uric acid level and prognosis of ischemic stroke, and other variables influencing the stroke were analyzed using Fisher's Exact Test. *P*-values less than 0.05 were considered significant.

## 3. Results

170 stroke patients were eligible to enter the study from which 145 were ischemic type (85.3%) and 25 were hemorrhagic (14.7%). 84 cases of ischemic stroke patients (57.9%) were thrombotic and the other 61 (42.1%) were of embolic origin. 18 cases of the hemorrhagic stroke patients (72%) were ICH and 7 (28%) were SAH. Demographic data of the patients along with their stroke types and risk factors are shown in Table 1. 57 ischemic stroke patients who had a history of high blood pressure were thrombotic and the other 27 cases were embolic, with a significant difference between the two subgroups (*P* = 0.004) (Table 1).

In diabetic stroke patients, 37 cases had thrombotic and 17 had embolic ischemic strokes (*P* = 0.047) (Table 1). There was no significant difference in the association between the subtypes of ischemic stroke with other risk factors such as ischemic heart disease, hyperlipidemia, and smoking (Table 1).

Considering the severity of stroke during admission in patients with ischemic stroke, 42 thrombotic cases had mild involvement, 36 had moderate involvement, and 6 had severe involvement. Among patients with embolic ischemic stroke, 40 had mild and 21 had moderate severity. The severity of the stroke at the time of admission and discharge in different types of stroke is shown in Table 1.

According to Table 2, of the total 170 included stroke patients, 57% had normal, 25% had low, and the remaining patients (18%) had high serum uric acid levels. As a result, the majority of stroke patients had normal serum uric acid levels.

Since the frequency of patients with hemorrhagic stroke was low in this study, statistical analyses could not be done on them. However, a significant portion of ICH patients had low levels of serum uric acid (Table 2).

There was no significant difference in serum uric acid levels in different types of strokes in both genders, as showed in Table 2.

Table 3 shows that, among the diabetic patients with a thrombotic ischemic stroke, 11 had low uric acid levels, 18 had normal, and 8 had high uric acid levels. These differences were not significant between the groups (*P* = 0.44). Contrary to this finding, in diabetic embolic ischemic patients, 4, 6, and 7 cases had low, normal, and high levels of uric acid, respectively, which showed a significant difference between the groups (*P* = 0.007). Diabetic embolic ischemic stroke cases had higher (SUA) levels than thrombotic types.

Thrombotic ischemic stroke patients with low magnesium levels had low, normal, and high levels of uric acid in 14, 18, and 6 cases, respectively (*P* = 0.030). Embolic ischemic stroke patients with low magnesium levels had low, normal, and high levels of uric acid in 8, 10, and 2 cases, respectively, showing a significant difference between the groups (*P* = 0.018) Table 4. Based on these findings, ischemic stroke (thrombotic and embolic) patients with low serum levels of magnesium had significantly lower serum uric acid levels compared with ischemic stroke patients with normal serum levels of magnesium.

Based on the findings, relationships between serum uric acid levels in serum Ca in thrombotic (*p* = 0.78), embolic (*p* = 0.63), and serum levels of Vit D in thrombotic (0.86) and embolic (0.23) were not significant in ischemic stroke patients.

We also found that serum uric acid levels were not significantly associated with the severity of ischemic stroke at the time of admission in thrombotic (*p* = 0.59) and embolic (*p* = 0.43) and at the discharge time in thrombotic (*p* = 0.38) and embolic (*p* = 0.41). Furthermore, we found no meaningful relationship between serum uric acid levels and stroke risk factors such as hypertension (thrombotic (0/18), embolic (*p* = 0.088)), ischemic heart disease (thrombotic (*p* = 0.531), embolic (*p* = 0.538)), hyperlipidemia (thrombotic (*p* = 0.71), embolic (*p* = 0.56)), and smoking (thrombotic (*p* = 0.49), embolic (*p* = 0.56)). Also relationships between serum uric acid levels and demographic variables, such as type of the living area (rural-urban), thrombotic (*p* = 0.48), and embolic (*p* = 0.99), were not significant.

Measured variables in this study were all investigated by logistic regression test in terms of independent effect on the risk of ischemic stroke (Table 5). By doing so, we found that the level of serum uric acid does not have any significant relationship with stroke (*P* = 0.807), although serum magnesium levels were identified as independent risk factors for ischemic stroke.

## 4. Discussion

The majority of stroke cases in this study were of ischemic type (85.3%). In these patients, the frequency of thrombotic ischemic stroke cases (57.9%) was higher than embolic subtypes (42.1%), but the relationship was only significant in hypertensive and diabetic ischemic stroke cases.

One of our general findings in this study was that the moderate severity of stroke at the time of admission and also at the time of discharge was higher in thrombotic ischemic stroke patients in comparison to embolic cases.

In our study, 50% of stroke patients had normal, 25% low, and the remaining patients (18%) had high serum uric acid levels, which was similar to the findings of many other studies [13, 14].

22% of ischemic stroke patients had low serum uric acid levels, while 17% of them had high levels. In another Iranian study, 47.3% of ischemic stroke patients were hyperuricemic [15]. This great difference between the two studies may be due to geographical differences which can significantly affect the results; one is performed in Tehran, a metropolitan city, whereas our study was performed in a small town, which has different lifestyles and diets.

According to logistic regression test, the level of serum uric acid in our studied stroke patients does not have any significant relationship with stroke.

The results of a relatively similar study conducted by Kawase et al. [16] were in line with ours.

In some other studies, although high serum uric acid level has been suggested as a risk factor for stroke, further studies were recommended for the final conclusion [17, 18].

Half of the ICH patients had serum levels of uric acid lower than normal. Contrary to this finding, not only a study by Wu et al. mentions no relationship between serum levels and ICH [19], but also as Ryu et al. reported high serum levels of uric acid and ICH has been strongly correlated especially in hypertensive cases [20].

The contradiction of results of relationship between serum levels and ICH in various studies shows that this issue should be investigated in future studies from different aspects. Based on results of this study, most ischemic stroke patients with mild to moderate severity at admission time had normal serum uric acid levels, while a review of the reports of other studies in this regard has controversial results. For example, findings of Brouns's study in 2010 showed that a decrease in serum levels of uric acid was associated with a greater intensity of stroke in the first 7 days after the stroke [5]. On the contrary, a report by Chiquete in 2013 indicated that a low concentration of serum uric acid was associated with good short-term outcomes in patients with stroke [21]. These contradictions also exist on the relationship of high serum uric acid levels and the severity of stroke.

Results of 2 studies by Amaro in 2011 and 2015 showed that increased serum uric acid levels were associated with better outcomes for stroke patients [22, 23].

Wu and colleagues also reported that uric acid could be a neuroprotective agent for acute ischemic stroke [19]. In their 2016 report, Wang et al. similarly concluded that serum uric acid has a neuroprotective effect in patients with ischemic stroke [24]. On the other hand, contrary to the findings suggesting a positive effect of serum uric acid level in the short-term prognosis of stroke, a study by Storhaug in 2013 [25] and some other studies [13] demonstrated that increased levels of uric acid could be a risk factor for ischemic stroke.

Our study suggested there was no significant relationship between the serum uric acid levels and the gender of stroke patients. This issue has been a topic of debate among researchers.

A case-control study by Jimenez et al. [26] was performed on 460 ischemic strokes in women, in which logistic regression analysis was used to eliminate the effects of age, race, smoking, menopause, the history of hypertension, and diabetes mellitus. They reported that serum levels of uric acid alone were not related to ischemic stroke [26], a finding similar to ours.

In Chongke Zhong's study, high serum uric acid levels were associated with increased risk of stroke in both men and women [27], while in the study by Storhaug, there was a significant difference between genders in terms of serum uric acid levels in ischemic stroke patients: they reported the level of uric acid was associated with a 31% increase in the risk of stroke in men [25] In a study by Kawase in 2017, although lower (SUA) level at the onset of ischemic stroke was associated with an increase in the incapacity of patients during the early hospitalization, this relationship was in the same way in both genders [16].

In another study to evaluate the relationship between SUA and ischemic stroke according to gender, it has been concluded that, in women with ischemic stroke who are treated with Alteplase, prescribing uric acid reduces the extent of the lesion and results are better than placebo [28]. As these studies have contradictory results, the relationship between uric acid levels and patients' gender cannot be conclusive.

The reason behind not finding a relationship between serum uric acid levels and gender of our stroke patients was not actually clear. Although the number of our studied patients was small, based on this study results, we could conclude that mechanism of possible effects of uric acid on stroke might be the same in both genders.

We observed higher levels of serum uric acid in our diabetic embolic ischemic stroke patients, compared with thrombotic cases. There is a lot of research on the relationship between serum uric acid levels and diabetes. In a case-control study, the mean (SUA) level in hypertensive and diabetic subjects was higher than that in normotensive and nondiabetic patients [29], which is generally in line with our findings.

Although (SUA) level in the prognosis of our diabetic ischemic patients had not any effect, Shuolin Wu showed that low SUA levels could predict the short-term poor functional outcome in acute stroke cases with normoglycaemia compared with diabetic or prediabetic cases [30]. While our study showed a difference between serum uric acid levels in ischemic thrombotic and embolic stroke only in diabetic patients, a report by Yang et al. suggested that serum uric acid (SUA) levels in acute ischemic stroke are generally associated with the risk of cardioembolic stroke [31].** Although embolic ischemic patients of our study included both of artery-to-artery embolisms and the cardioembolisms, it seems that according to the findings of these two studies and some other studies, there is a relationship between serum (SUA) level and acute artery-to-artery or cardioembolic ischemic stroke in diabetics, which should be further investigated to confirm this relationship and its exact type**.

In our study, ischemic stroke patients with low serum levels of magnesium had significantly lower amounts of serum uric acid compared with those having normal or high serum levels of magnesium. The cause or causes of this finding and its significance are not clear to us at this time.

However, the relationship between magnesium and stroke has been extensively studied. In a study by Adebamovo et al. increasing the consumption of dietary magnesium caused a decline in the incidence of stroke, especially in women [32]; in another study, lower plasma magnesium levels were associated with higher risk of ischemic stroke among women [33].

To confirm the clinical value of the our results about possible correlation between serum levels of magnesium and uric acid in stroke, more studies should be conducted with higher sample sizes.

There are studies on the relationship between uric acid and vitamin D in general, such as the one by Peng et al. in 2013, in which they reported vitamin D deficiency was significantly associated with an increase in serum levels of uric acid in postmenopausal women [34]. Our findings, however, indicate no significant relationship between uric acid and vitamin D and calcium in stroke. It is also worth noting that our study showed no meaningful relationship between serum uric acid levels and stroke risk factors such as hypertension, ischemic heart disease, hyperlipidemia, and smoking. In addition, we did not find a significant link between serum uric acid levels and some other associated factors such as type of the place of residence (rural-urban), which might had different (SUA) levels depending on the different lifestyle and diet in the two communities.

Although other factors such as BMI, dietary habits, physical exercise, and aspirin tablet use (which have a close relation with the serum uric acid level) were not determined, but considering the locality of the studied area and the cultural similarities of our patients, these factors may not have a significant impact on our results. However, we agree that these were some of the limitations of our study.

Small sample size of studied patients and lack of control group might have led to loss of the power statistical analysis and lack of long-term follow-up of cases was other limitations of this study. N**ondifferentiation of various types of embolic stroke, despite the different impacts from uric acid levels between cardioembolic and artery-to-artery embolic strokes, is a limitation of study.**

Investigating the relationship between (SUA) levels and stoke severity at admission and discharge time and the relationship between (SUA) levels and stroke risk factors and some associated factors such as type of the place of residence (rural-urban) and finally investigating the relationship between serum levels of uric acid and elements such as calcium and magnesium and hormones such as Vit D in stroke patients were the strengths of this study.

## 5. Conclusion

SUA level is not associated with stroke types and gender of patients. There are high SUA levels in diabetic embolic ischemic stroke cases and in ischemic stroke patients with low serum levels of magnesium; SUA levels are lower. There is no relationship between SUA levels and stroke risk factors such as hypertension, ischemic heart disease, hyperlipidemia, and smoking and some other associated factors such as type of living area (rural-urban).

There is no relationship between (SUA) levels and serum levels of vitamin D and calcium in stroke. Serum magnesium levels were identified as independent risk factors for ischemic stroke

## Figures and Tables

**Table 1:** Frequency of stroke patients by gender, risk factors, and severity of stroke.

Variable		Ischemic stroke (%)		*P*-value		Hemorrhagic stroke (%)	
		thrombotic	Embolic				
						ICH	SAH
Gender	male	(58.2) 39	(41.8)28	0.202		(80)12	(20)3
	female	(57.7)45	(42.3)33			(60)6	(40)4

hypertension	yes	(67.9)57	(32.1)27	0.004		(100)16	( 0) 0
	no	(44.3)27	(55.7)34			(22.2)2	(77/8)7

Ischemic Heart Disease	yes	(50)31	(50)31	0.999		(100)5	(0)0
	no	(63.9)53	(36.1)30			(65)13	(35)7

Diabetic disease	no	(63.9)53	(36.1)30	0.047		(90)9	(10)1
	yes	(68.5)37	(31.5)17			(60)9	(40)6

Diabetes mellitus	no	(51.6)47	(48.4)44	0.426		(60)9	(40)6
	yes	(54.1)33	(45.9)28			(100)3	(0)0

hyperlipidemia	yes	(54.1)33	(45.9)28	0.426		(100)3	(0)0
	no	(60.7)51	(39.3)33			(68.21)5	(31.8)7

smoking	Yes	(56.8)21	(43.2)16	0.867		(81.8)9	(18.2)2
	no	(58.3)63	(41.7)45			(64.3)9	(35.7)5

severity in admission time	mild	(50)42	(95.23)40	0.03	0.002	(16.66)3	(85.71)6
	moderate	(42.85)36	(4.7)2			(72.22)13	(14.29)1
	severe	(14.28)6	(0)0			(11.12)2	(0)0

severity in discharge time	mild	(58.75)47	(83.33)50	-	-	(90)9	(83.34)5
	moderate	(26.25)21	(16.67)10			(10)1	(0)0
	severe	(15)12	(0)0			(0)0	(16/66)1

**Table 2:** Serum uric acid levels by gender in stroke patients.

variable		Serum uric acid levels (%)				*P*-value
Stroke subtypes		gender	low	normal	High	
Ischemic	thrombotic	Male	(50)10	(41.2)21	(61.5)8	0.39
		Female	(50)10	(58.8)30	(38.5)5	
	embolic	Male	(41.7)5	(47.4)18	(45.5)5	0.94
		Female	(58.3)7	(52.6)20	(54.5)6	

Hemorrhagic	ICH	Male	(66.7)6	(60)3	(75)3	-
		Female	(33.3)3	(40)2	(25)1	
	SAH	Male	(0)0	(33.33)1	(50)1	-
		Female	(100)11	(66.67)2	(50)1	

**Table 3:** Serum uric acid levels by diabetes mellitus in stroke patients.

variable		Diabetes mellitus	Serum uric acid levels (%)			*P*-value
stroke subtypes			low	normal	high	
ischemic	thrombotic	yes	(55)11	(35.3) 18	(61.5)8	0.12
		no	(4) 9	(64.7) 33	(38.5)5	
	embolic	yes	(33.3) 4	(15.8) 6	(63.6)7	0.007
		no	(66.7) 8	(84.2)32	(36.4)4	

hemorrhagic	ICH	yes	(55.6) 5	(40) 2	(50)2	-
		no	(44.4) 4	(60) 3	(50)2	
	SAH	yes	(0) 0	(16.7)1	(0) 0	-
		no	(100) 1	(83.3) 5	(0)0	

**Table 4:** Serum uric acid levels by serum magnesium level in stroke patients.

variable		Serum magnesium level	serum Uric acid levels (%)			*P*-value
stroke subtypes			low	normal	high	
ischemic	thrombotic	low	(70) 14	(35.3) 1	(46.2) 6	0.030
		normal	(30) 6)	(64.7) 33	(53.8) 7	
	embolic	low	(66.7) 8	(26.3) 10	(18.2) 2	0.018
		normal	(33.3)4	(73.7) 2	(81.8) 9	

hemorrhagic	ICH	low	(77.8)7	(100) 5	(50 )2	-
		normal	(22.2) 2	(0) 0	(50 )2	
	SAH	low	(100) 1	(83.3) 5	(0 )0	-
		normal	(0) 0	(16.7) 1	(0 )0	

**Table 5:** The risk of ischemic stroke with adjusted variables by logistic regression test.

Variable	OD Ratio	% 95CI	*P*- value
gender	0.71	0.18–2.08	0.623
age	1.04	0.99–1.09	0.089
Living area	0.69	0.23–2.06	0.503
Mild stroke severity at admission	1.00	-	0.051
moderate stroke severity at admission	0.25	0.07–0.86	0.027
severe stroke severity at admission	0.11	0.01–0.98	0.048
hypertension	1.29	0.37–4.47	0.690
Ischemic heart disease	4.38	1.29–14.87	0.018
diabetes mellitus	1.55	0.49–4.97	0.458
hyperlipidemia	5.47	1.25–24.07	0.024
smoking	0.65	0.16–2.62	0.548
serum magnesium level	3.97	1.21–12.99	0.023
serum vitamin D level	2.27	0.65–7.95	0.199
serum calcium level	1.99	0.60–6.60	0.259
Low serum uric acid level	1.00	-	0.503
Normal serum uric acid level	1.99	0.62–6.38	0.248
High serum uric acid level	1.72	0.33–8.89	0.520
